# Surgical management of intraocular lens dislocation: A meta-analysis

**DOI:** 10.1371/journal.pone.0211489

**Published:** 2019-02-20

**Authors:** Shangfei Yang, Kailai Nie, Hui Jiang, Liwen Feng, Wei Fan

**Affiliations:** Department of Ophthalmology, West China Hospital of Sichuan University, Chengdu, Sichuan Province, China; Aston University School of Life and Health Sciences, UNITED KINGDOM

## Abstract

**Purpose:**

To compare the efficacy and safety of intraocular lens (IOL) repositioning and IOL exchange for the treatment of patients with IOL dislocation.

**Methods:**

We systematically searched for relevant publications in English or Chinese in MEDLINE, Embase, the Cochrane Central Register of Controlled Trials, WHO International Clinical Trial Registration Platform, Clinical Trial.gov, China Biology Medicine Database, China National Knowledge Infrastructure Database and grey literature sources. Study quality was assessed using the STROBE template for observational studies and the Cochrane template for randomized controlled trials (RCTs). Data were meta-analyzed using RevMan 5.3.

**Results:**

The review included 14 English-language studies reporting 1 RCT and 13 retrospective case series involving **1,082** eyes. Average follow-up time was 13.7 months. Pooled analysis of 10 studies showed that the two procedures had a similarly effect on best corrected visual acuity (MD -0.00, 95%CI: -0.08 to 0.08, P = 0.99). Pooled analysis of nine studies showed no significant difference in incidence of IOL redislocation (RR 2.12, 95%CI 0.85 to 5.30, P = 0.11); pooled analysis of seven studies showed greater extent of incidence of cystoid macular edema in IOL exchange (RR 0.47, 95%CI 0.21 to 1.30, P = 0.06). Pooled analysis of three studies showed greater extent of incidence of anterior vitrectomy in IOL exchange (RR 0.11, 95%CI 0.04 to 0.33, P<0.0001). Pooled analysis of two studies showed greater postoperative spherical equivalents in IOL repositioning (MD 1.02, 95%CI 0.51 to 1.52, P<0.0001). pooled analysis suggested no significant differences between the two procedures in terms of intraocular pressure, endothelial cell density, surgically induced astigmatism, or incidence of retinal detachment, intraocular hemorrhage or pupillary block.

**Conclusion:**

IOL repositioning and exchange are safe and effective procedures for treating IOL dislocation. Neither procedure significantly affects best corrected visual acuity and IOL redislocation. IOL exchange was superior to repositioning in terms of postoperative SE, but IOL repositioning was associated with lower incidence of anterior vitrectomy, potentially lower incidence of cystoid macular edema.

## Introduction

Intraocular lens (IOL) dislocation is an uncommon but serious postoperative complication of cataract surgery, with incidence ranging from 0.2–3% [1–3]. Actual incidence rate may be even higher, due to the large numbers of patients who have undergone cataract surgery and the increasing trend in IOL dislocation cases in recent years [4–6]. Posterior chamber IOL dislocation in the early postoperative period, especially the first 3 months, occurs usually outside of the capsule. The main risk factors associated with such dislocation are asymmetrical fixation and intraoperative complications, especially in complicated cataract surgery [5–8]. In-the-bag IOL dislocation occurs usually several years after cataract surgery, primarily as a result of zonular weakness and inadequate capsule. These two risk factors usually arise from pseudoexfoliation syndrome, myopia/increased axial length, Nd:YAG capsulotomy, vitreoretinal surgery, retinitis pigmentosa, trauma, uveitis and certain connective tissue disorders [5,7,9–13].

When IOL dislocation is limited and does not affect vision acuity, most clinicians advocate conservative treatment. In contrast, if the edge of the IOL can be seen in the pupil area in the absence of pupil dilation, vision acuity may be seriously affected and the IOL dislocation must be treated [14]. For out-of-the-bag IOL dislocation during the early postoperative period, repositioning into the capsular bag or ciliary sulcus without suturing is the preferred treatment. In contrast, two surgical approaches may be used to treat complicated out-of-the bag IOL dislocations and late in-the-bag IOL dislocations. One is repositioning the existing IOL, by fixating it either to the scleral or to the iris. Another is replacing the dislocated IOL with a new anterior chamber IOL or posterior chamber IOL by fixating it either to the scleral or to the iris, or to the ciliary sulcus if there is sufficient capsule [7,14,15].

Optimal management for IOL dislocation remains controversial. Several studies have compared various procedures [5,7,9–11,15–25], but the conclusions are conflicting. Here we examined the available literature to compare the efficacy and safety of IOL repositioning and IOL exchange for treating IOL dislocation.

## Patients and methods

### Registration

The review was registered on PROSPERO of the Centre for Reviews and Dissemination (CRD42018075934).

### Inclusion and exclusion criteria

We included studies in our review and meta-analysis (1) if they were randomized controlled trials (RCTs) or observational studies comparing IOL repositioning and IOL exchange, either alone or in combination with vitrectomy, for the treatment of IOL dislocation; (2) if patients were diagnosed with IOL dislocation based on slit lamp examination; and (3) if the study was published in English or Chinese. Studies were excluded if they were reviews, reports of laboratory findings only, or trials published only as abstracts.

### Comparisons and outcome measures

IOL repositioning was compared with IOL exchange in terms of primary and secondary outcomes. The primary outcomes were best corrected visual acuity (BCVA), incidence of IOL redislocation, incidence of cystoid macular edema (CME), incidence of anterior vitrectomy and spherical equivalents (SE). Secondary outcomes were intraocular pressure (IOP), surgically induced astigmatism(SIA), endothelial cell density (ECD), and incidence of retinal detachment, intraocular hemorrhage, and pupillary block.

### Data resources and searches

A systematic search was performed to MEDLINE (1966–2018.6), Embase (1947–2017.9), Cochrane Central Register of Controlled Trials (1948–2018.6), WHO International Clinical Trial Registration Platform (ICTRP) (2004–2018.6), Clinical Trial.gov (1999–2017.9), China Biology Medicine disc (1978–2018.6) and China National Knowledge Infrastructure (1979–2018.6). The included trials' references were searched for more studies; experts in the field were consulted. The language was restricted to English and Chinese.

To ensure detection of as many potentially relevant studies as possible, we did not limit the types of study design or interventions. The following sequence of searches was performed on OVID:

#1 exp Lenses, Intraocular/

#2 Lens Implantation, Intraocular/

#3 (intraocular or intra ocular or intra-ocular or lens$ or IOL$).tw.

#4 Lens, Intraocular.tw.

#5 Intraocular Lens.tw.

#6 Implantable Contact Lens.tw.

#7 Contact Lens, Implantable.tw.

#8 Lens, Implantable Contact.tw.

#9 1 or 2 or 3 or 4 or 5 or 6 or 7 or 8

#10 dislocation.tw.

#11 Subluxation.tw.

#12 luxation.tw.

#13 10 or 11 or 12

#14 9 and 13

### Study selection and data extraction

Two reviewers independently assessed the titles and abstracts of the searched results. Full texts of potentially eligible studies were then screened to identify the final included studies. For each included study, following information were extracted: name of study, acceptance date(year), study design, selection criteria, participant's characteristics, interventions, outcome measures, study duration, follow up duration, results, and other data. Disagreements could be resolved by discussion.

LogMAR data on visual acuity were extracted from studies. If studies reported visual acuity in other units, the values were transformed to LogMAR as described [26].

### Risk of bias assessment

The quality of included studies was assessed using the Cochrane template for randomized controlled trials (RCTs) [27] or the STROBE template for observational studies [28–29] in the case of non-randomized studies by two reviewers independently. Non-randomized studies with a score of 15 or more were classified as high-quality studies [30]. Publication bias was assessed with Begg’s funnel plot and Egger’s test using Stata 12.0.

### Data synthesis and analysis

Data were meta-analyzed using RevMan 5.3 (Cochrane Collaboration). We used a random-effect model to calculate pooled mean differences (MDs)for continuous data and risk ratios (RRs) for dichotomous data, together with 95% confidence intervals. Transformation was undertaken of initial data for entry into RevMan5.3 when necessary. Heterogeneity was assessed using the χ^2^ test and I^2^ value, and either χ^2^ P value <0.10 or I^2^>50% was considered that heterogeneity was significant. Potential sources of heterogeneity were investigated by subgroup analyses (study setting, surgical techniques and ethnic origin), and sensitivity analyses. The threshold for significance was defined as P = 0.05. [31]

## Results

### Characteristics of included studies

**Fig 1:**
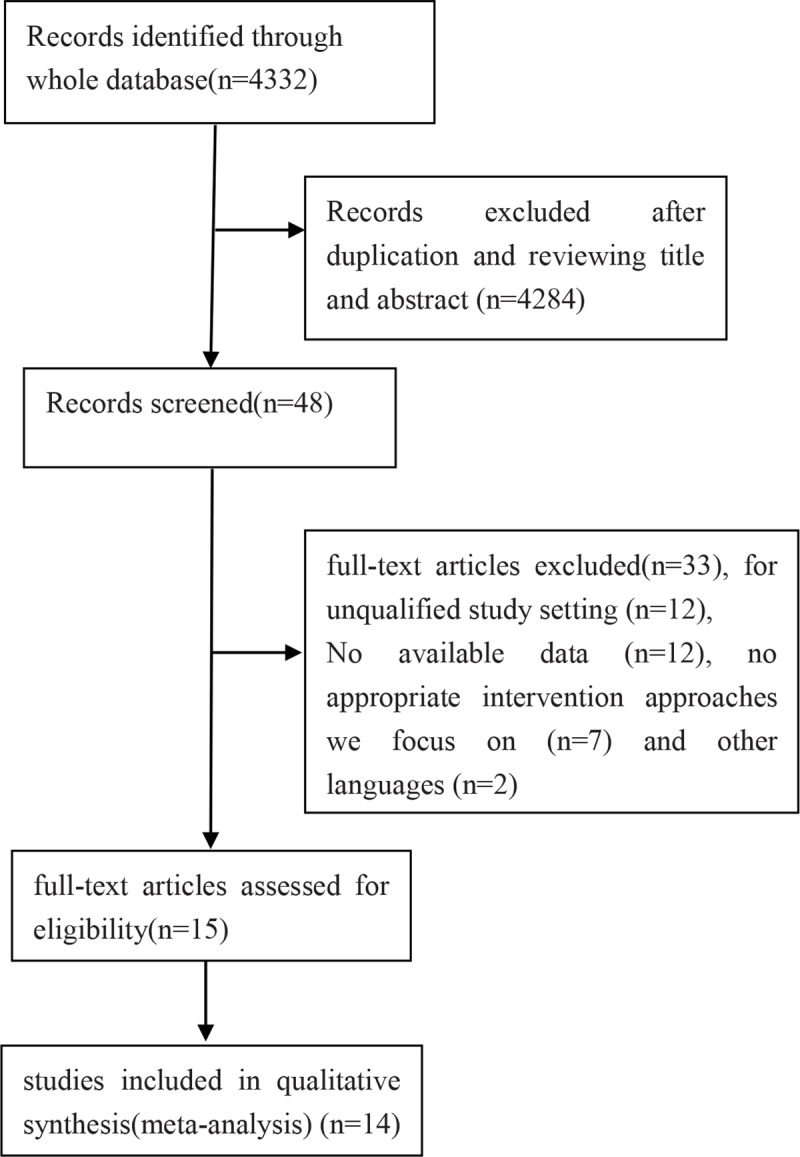
Flow diagram of study selection.

A total of 4,332 articles were identified in database and manual searches, and 48 articles were read in full after duplicates and irrelevant studies had been excluded based on review of titles and abstracts. On the basis of full-text review, 12 studies were excluded because the study design was inappropriate, 12 because they did not report available data on the primary or secondary outcomes, 7 because they did not involve the target interventions and 2 because they were not published in English or Chinese (**Fig 1**). In the end, 14 studies involving 1,082 eyes were analyzed, including one RCT and 13 retrospective case series. The baseline characteristics of the included studies are summarized in **Table 1**.

**Table 1:** Characteristics of included studies.

Study	Design	Location	Age	Patients	Male	Female	Eyes	studying time(m)	Duration from CS to IOLD(y)	Follow up (m)	Type of IOLD	Predisposing conditions	Repositioning		Exchange		Quality
													Technique	Eyes	Technique	Eyes	
Oh2015 [7]	RCS	Korea	62.2	25	18	7	25	27	Unclear	6	ITB/OTB	Trauma/ Capsulotomy	Fibrin glue-assisted sutureless scleral	13	A new IOL by fibrin glue- assisted sutureless scleral	12	15.5
Gross2004 [9]	RCS	USA	74.9	22	15	7	25	120	6.9	12.3	ITB	PEX/Uveitis/Trauma	Suture-Scleral	7	ACIOL/Suture-Scleral	18	10
Ganesh2017 [10]	RCS	India	51	6	3	3	7	215	11.2	23.8	ITB	Uveitis	Suture-Scleral	2	Suture-Scleral	5	13.5
Gul2015^[^^11^^]^	RCS	Turkey	55.6	26	15	11	28	26	3.2	13.3	ITB/OTB	PEX/Capsular rupture /Trauma/High myopia	Suture-Scleral/nosuture-sulus	15	Suture-Scleral/nosuture-sulus/bag	11	12
Kristianslund2017 [14,16,17]	RCT	Oslo	81.7	104	41	63	104	35	10.3	6	ITB	PEX/Myopia/ VR Surgery /Trauma/Chronic uveitis	Suture-Scleral	54	IC IOL	50	High
Shingleton2013 [15]	RCS	USA	83.8	81	28	53	81	265	8.5	30	ITB/OTB	PEX	Suture-Scleral/iris	17	ACIOL/Suture-Scleral/iris	64	15.5
Ostern2014 [18]	RCS	Norway	82	77	32	45	81	73	8.5	15.5	ITB	Trauma/Glaucoma surgery/ Capsulotomy/VR surgery	Suture-Scleral	50	ACIOL/ IC IOL/ Suture-Scleral	23	14.5
Lorente2010 [19]	RCS	Spain	80.7	41	23	18	44	50	8.4	13.6	ITB	Glaucoma surgery/PEX/VR surgery/Capsulotomy	Suture-Scleral/iris	21	ACIOL/IC IOL	23	13
Kim2008 [20]	RCS	USA	72.3	277	130	147	284	221	2.8	17.4	ITB/OTB	VR surgery/Capsulotomy/PEX	ACIOL/Suture-Scleral/nosuture-sulus	224	ACIOL/Suture-Scleral/nosuture-sulus	73	14
Sarrafizadeh2001 [21]	RCS	USA	79.1	56	28	28	59	89	Unclear	34	ITB/OTB	Trauma/Capsulotomy	Suture-Scleral/nosuture-sulus	29	ACIOL/Suture-Scleral	30	15
Smiddy2005 [22]	RCS	USA	72	32	16	16	32	84	0.3	9.3	Unclear	Retained lens fragments /PEX	Suture-Scleral/nosuture-sulus	25	ACIOL/Suture-Scleral/nosuture-sulus	7	10.5
Schneiderman1997 [23]	RCS	USA	71.4	11	4	7	11	unclear	0.2	6.5	Unclear	Capsulotomy/ Capsular rupture at cataract surgery	Nosuture-sulus	6	ACIOL/Suture-Scleral/nosuture-sulus	5	9.5
Smiddy1995 [24]	RCS	USA	74	78	44	34	78	24	10	6.5	Unclear	Glaucoma/PEX/High myopia/Marfan's syndrome	Suture-Scleral/nosuture-sulus	43	ACIOL/Suture-Scleral/nosuture-sulus	29	10
Baba [25]	RCS	Japan	65	15	-	-	15	14	8.3	Unclear	ITB	Unclear	Nonsuture-Scleral	6	Nonsuture-Scleral	9	9.5

CS: cataract surgery; IOLD: intraocular lens dislocation; RCT: randomized controlled trial; RCS: retrospective case series; ITB; in-the-bag; OTB: out-of-the-bag; PEX; Pseudoexfoliation syndrome; VR; Vitreoretinal; IC IOL: iris-claw intraocular lens; ACIOL: anterior chamber intraocular lens

The average age of patients was 73.6±10.1yr, and average duration of follow-up was 13.7 months. Quality assessment of the RCT indicated a low risk of bias [14,16,17]. Three studies received a score≥15 [7,15,21]; 8 studies, a score of 10–15 [9–11,18–20,22,24]; and 2 study, a score <10 [23,25].

### Meta-analysis

Assessment of heterogeneity revealed a P value >0.1 for the χ^2^ test and I^2^ < 50%. Therefore, we used a random-effect model to calculate pooled mean differences (MDs) and risk ratios (RRs).

**Fig 2:**
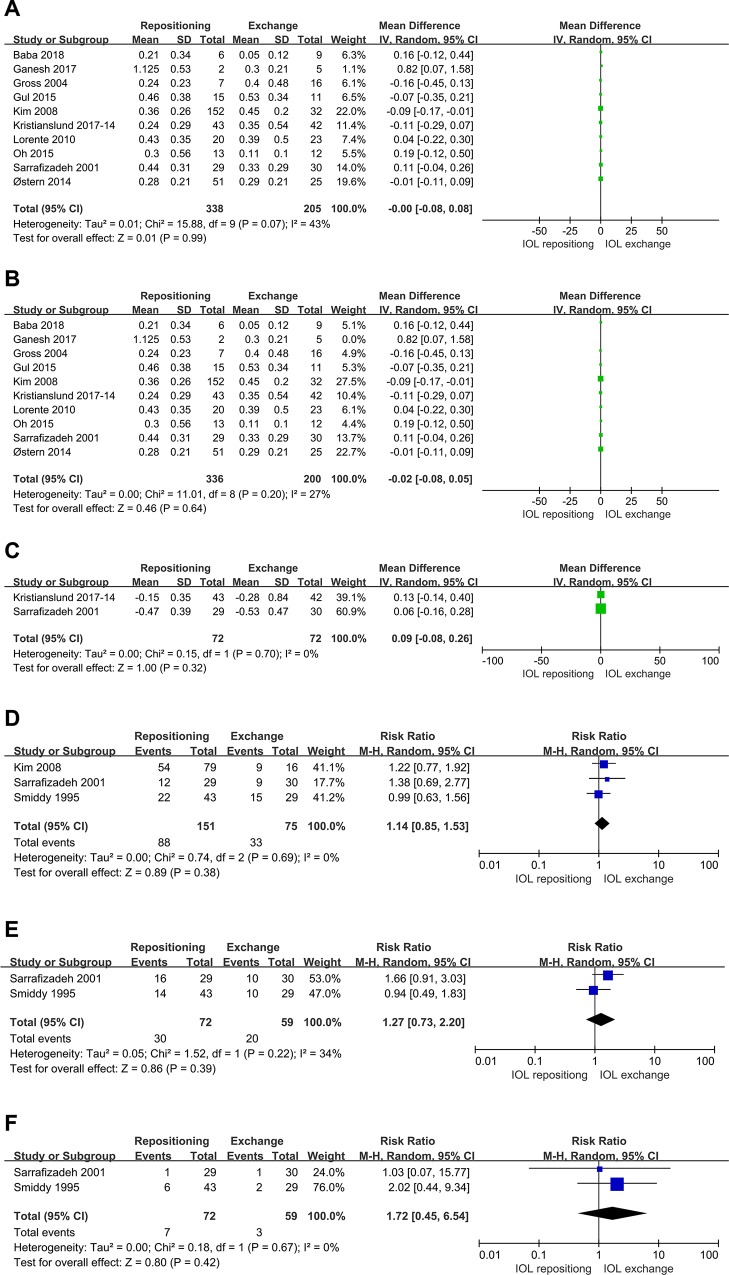
Comparison of IOL repositioning and IOL exchange in terms of BCVA. (A) Postoperative BCVA. (B) Sensitivity analysis of postoperative BCVA, after excluding a small study. (C) Difference between peri- and postoperative BCVA. (D) Incidence of BCVA>20/40. (E) Incidence of BCVA 20/50–20/200. (F) Incidence of BCVA <20/200.

Ten studies compared IOL repositioning and exchange for the primary outcome of postoperative BCVA. Pooled analysis showed that the two procedures had a similar effect on BCVA (MD -0.00, 95%CI: -0.08 to 0.08, P = 0.99; **Fig 2A**). We obtained similar results after excluding one small study [10] (**Fig 2B**). Pooled analysis of the two studies reporting the change in BCVA as a result of surgery showed the two procedures to be similar (MD 0.09, 95%CI -0.08 to 0.26, P = 0.32; **Fig 2C**). Some studies treated postoperative BCVA as a categorical rather than continuous variable; again, the two procedures showed similar results: BCVA>20/40, RR1.14 (95%CI 0.85 to 1.53; P = 0.38; **Fig 2D**); BCVA 20/50–20/200, RR 1.27 (95%CI 0.73 to 2.20;P = 0.39; **Fig 2E**); and BCVA<20/200, RR 1.72 (95%CI 0.45 to 6.54; P = 0.42; **Fig 2F**).

**Fig 3:**
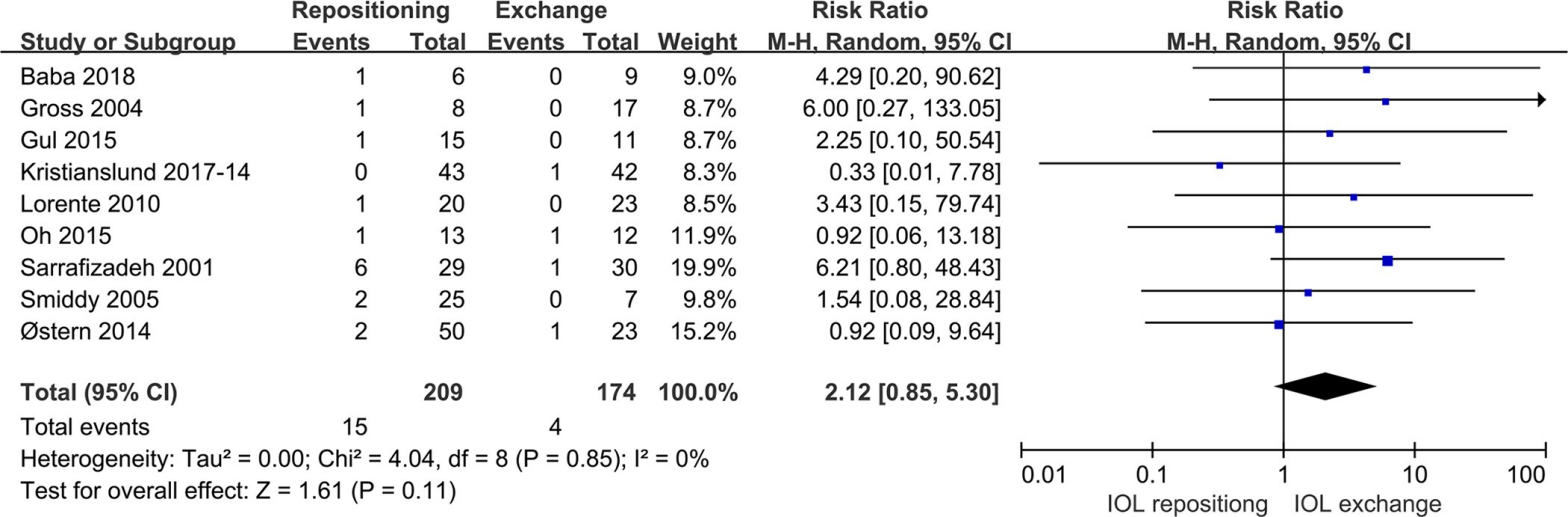
Comparison of IOL repositioning and IOL exchange in terms of incidence of IOL redislocation.

Nine studies compared IOL repositioning and exchange for the primary outcome of IOL redislocation. Pooled analysis showed no significant difference in IOL redislocation between the two procedures (RR 2.12, 95%CI 0.85 to 5.30, P = 0.11; **Fig 3**).

**Fig 4:**
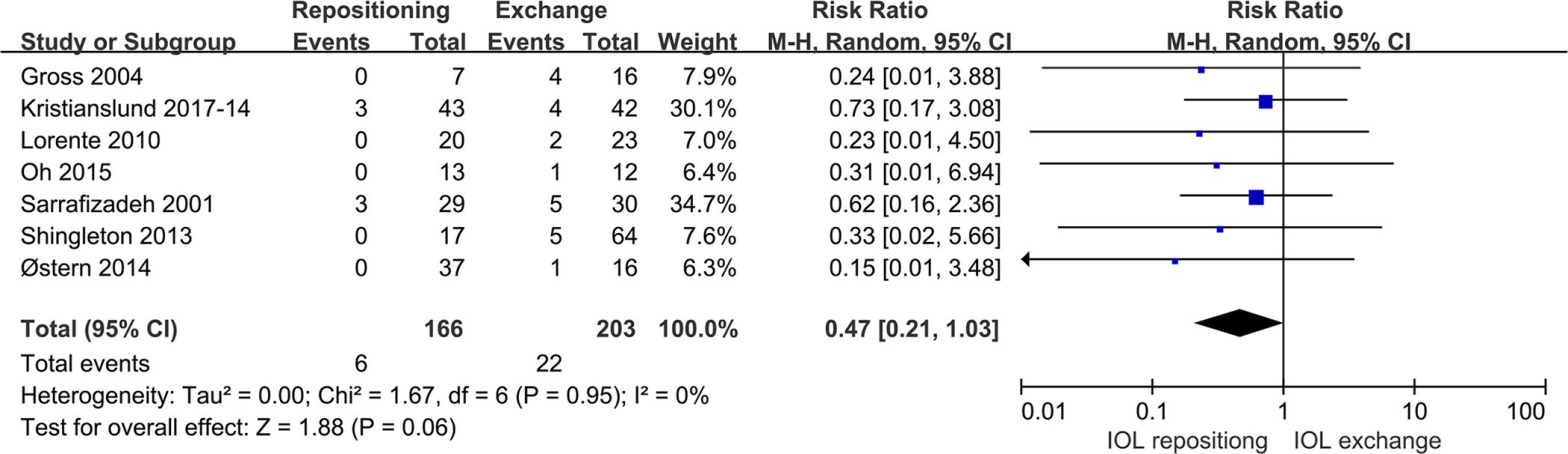
Comparison of IOL repositioning and IOL exchange in terms of CME incidence.

Seven studies compared the safety of the two surgical approaches in terms of CME incidence. Pooled analysis showed no significant difference in the term between the two procedures (RR 0.47, 95%CI 0.21 to1.03, P = 0.06; **Fig 4**).

**Fig 5:**
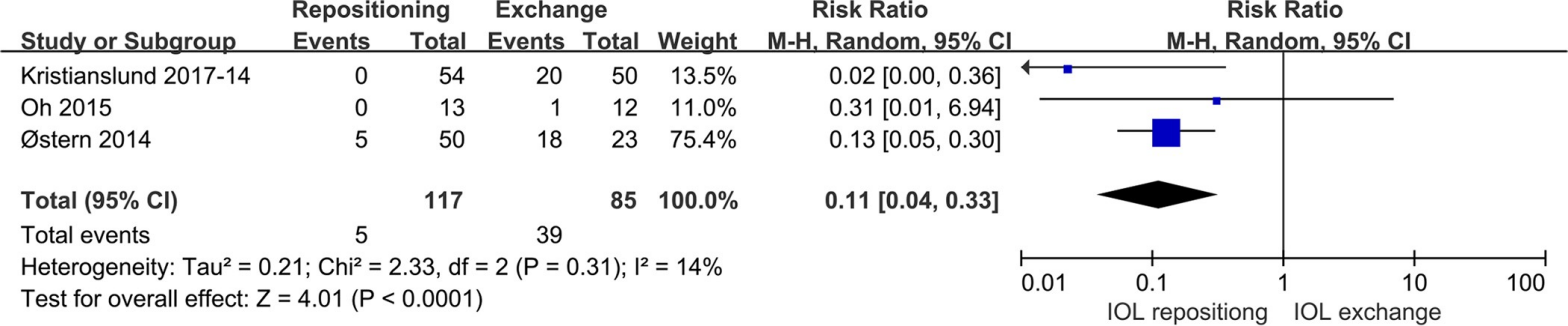
Comparison of IOL repositioning and IOL exchange in terms of incidence of anterior vitrectomy.

For another primary safety outcome, the incidence of anterior vitrectomy, we found that IOL repositioning showed lower incidence of anterior vitrectomy than IOL exchange (RR0.11, 95%CI 0.04 to 0.33, P<0.0001; **Fig 5**).

**Fig 6:**
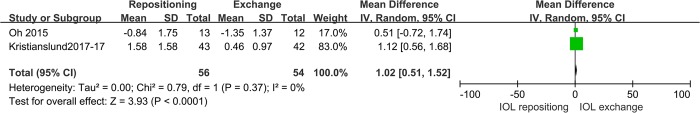
Comparison of IOL repositioning and IOL exchange in terms of postoperative SE.

For the outcome of postoperative SE, IOL exchange showed significant superiority over IOL repositioning. Pooled analysis showed MD 1.02 (95%CI 0.51 to 1.52, P<0.0001; **Fig 6**).

**Fig 7:**
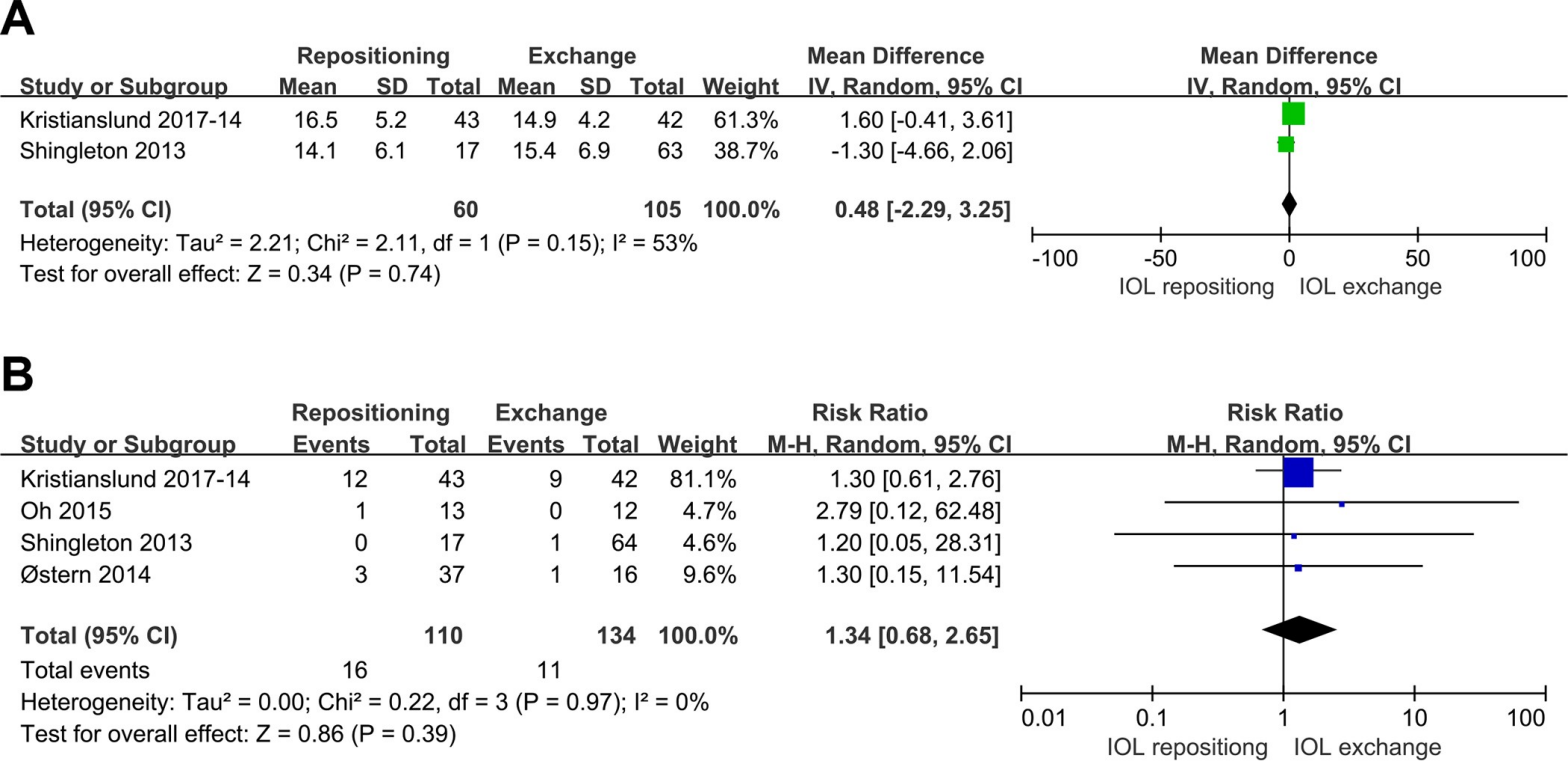
Comparison of IOL repositioning and IOL exchange in terms of IOP. (A) Postoperative IOP. (B) Increase in postoperative IOP relative to perioperative IOP.

Two studies compared IOL repositioning and exchange for the outcome of postoperative IOP. Pooled analysis showed no significant difference in IOP between the two procedures (MD 0.48, 95%CI: -2.29 to 3.25, P = 0.74; **Fig 7A**). Pooled analysis of four studies reporting the incidence of higher IOP after surgery showed similar results for the two surgical approaches (RR 1.34, 95%CI 0.68 to 2.65, P = 0.39; **Fig 7B**).

**Fig 8:**
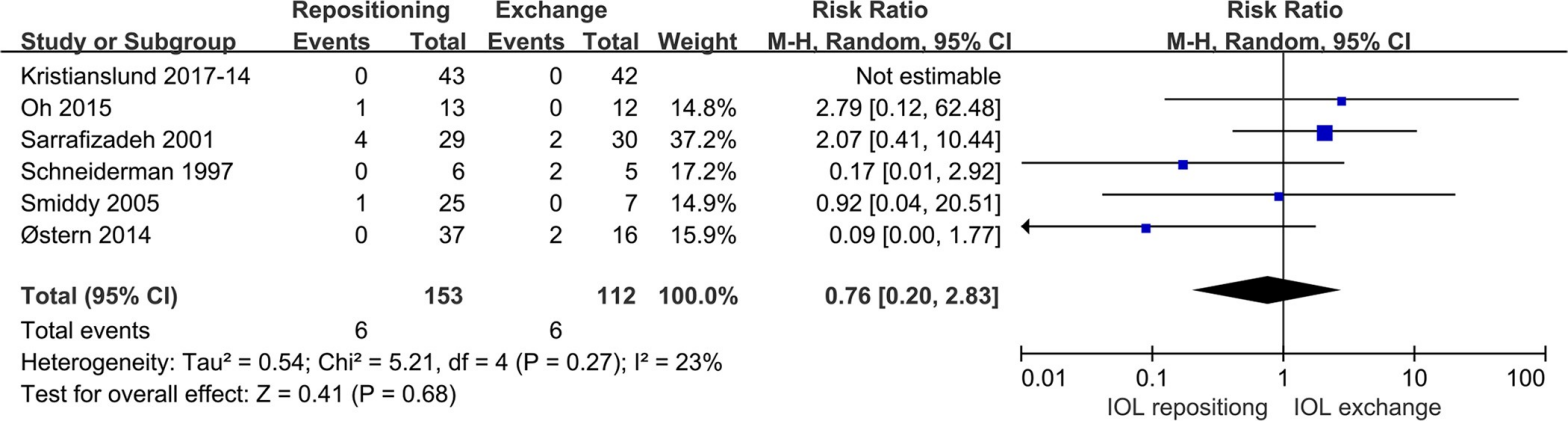
Comparison of IOL repositioning and IOL exchange in terms of incidence of retinal detachment.

Six studies compared IOL repositioning and exchange for the outcome of retinal detachment. Pooled analysis showed no significant difference in the term between the two procedures (RR 0.76, 95%CI0.20 to 2.83, P = 0.68; **Fig 8**).

**Fig 9:**
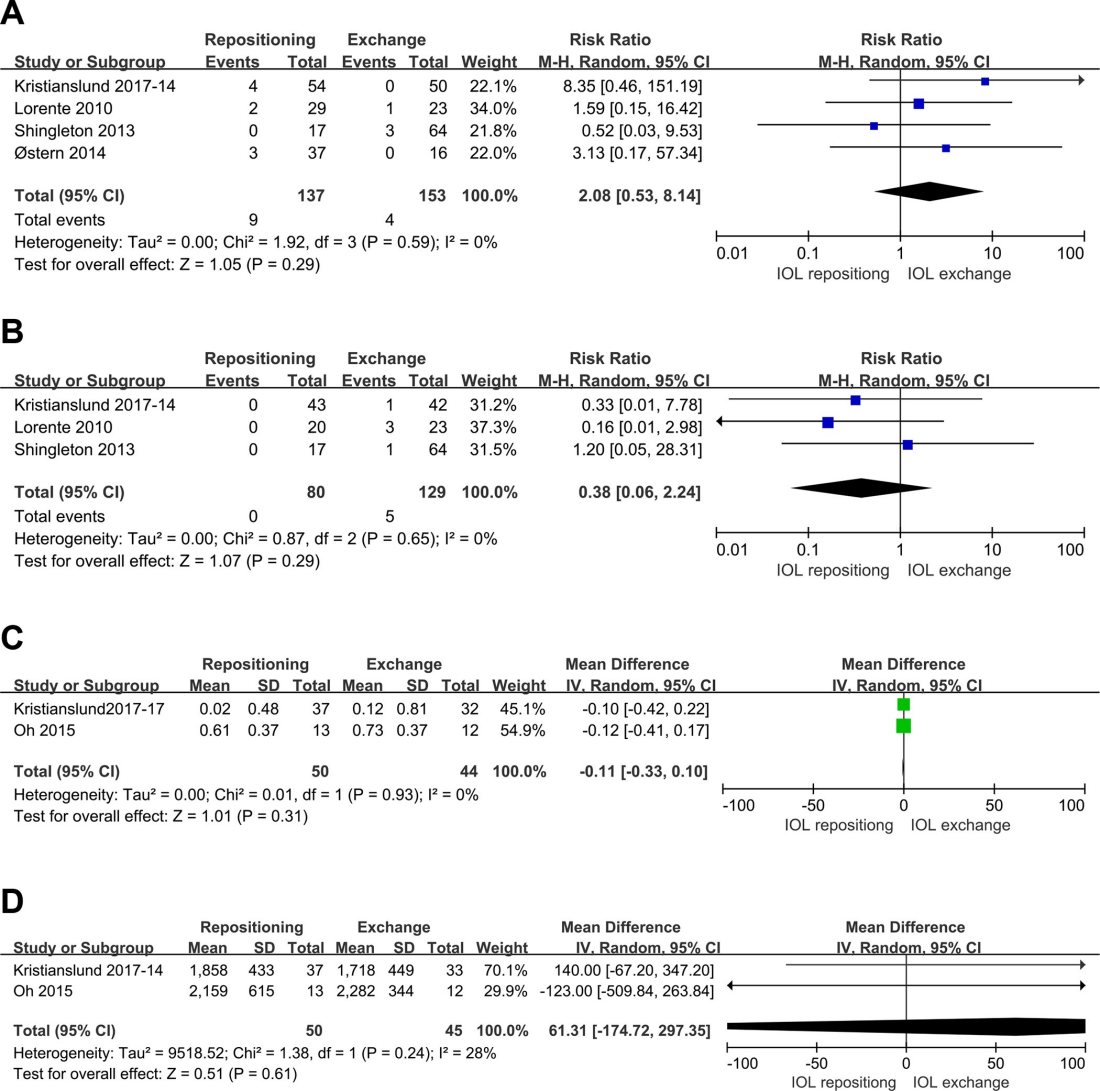
**Comparison of IOL repositioning and IOL exchange in terms of other complications:**(A) incidence of intraocular hemorrhage; (B) incidence of pupillary block; (C) SIA; (D) postoperative ECD.

Pooled analysis revealed no significant differences between IOL repositioning and exchange in several other complications: incidence of intraocular hemorrhage and pupillary block, SIA and postoperative ECD (P>0.05) (**Fig 9**).

Some primary and secondary outcomes were re-analyzed using data only from the retrospective case series (Table 2). The results were consistent with the pooled analysis of retrospective case series and the RCT.

**Table 2:** Pooled analysis of retrospective case series only.

Outcomes	Number of studies	MD (or RR)	95% CI	P-value for difference	Residual heterogeneity (tau2)
BCVA	9	0.02	-0.07, 0.11	0.73	0.01
Redislocation	8	2.52	0.97, 6.55	0.06	0.00
CME	6	0.39	0.15, 0.09	0.05	0.00
Anterior vitrectomy	2	0.14	0.06, 0.31	<0.00001	0.00
Higher IOP	3	1.54	0.33, 7.31	0.59	0.00
Retinal detachment	5	0.76	0.20, 2.83	0.68	0.54
Intraocular hemorrhage	3	1.40	0.30, 6.58	0.67	0.00
Pupillary block	2	0.42	0.05, 3.46	0.41	0.00

**Fig 10:**
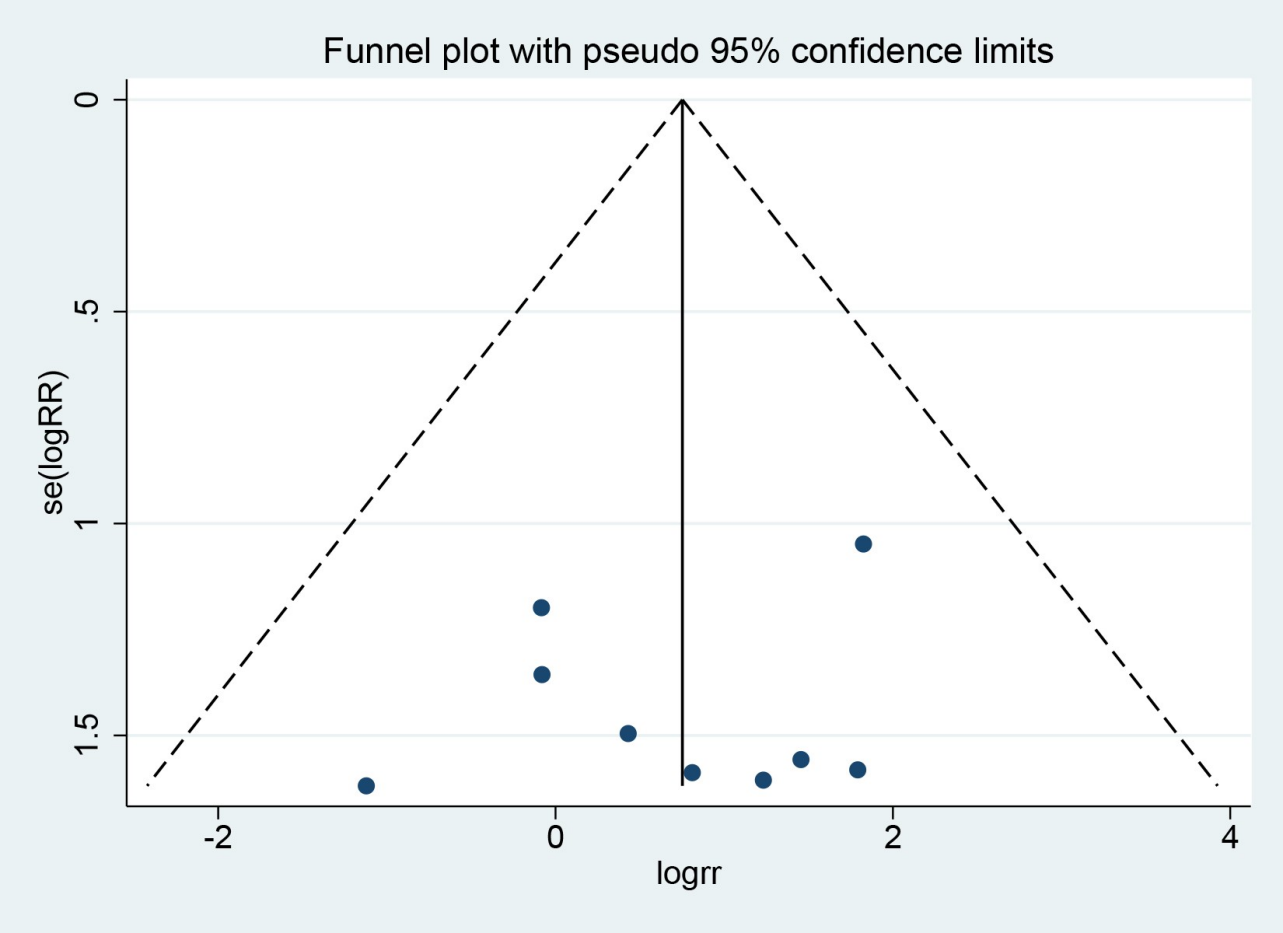
Funnel Plot of Publication Bias for IOL redislocation.

Publication bias was evaluated using Begg’s funnel plot and Egger’s test. The funnel plot comparing IOL repositioning and exchange in terms of incidence of IOL redislocation appeared symmetrical (Begg’s test P = 0.917). Egger’s test suggested no significant risk of publication bias (Egger’s test P = 0.554) (**Fig 10**).

## Discussion

IOL dislocation is a rare but serious complication after cataract surgery. It can seriously affect visual acuity, and it can lead to other serious complications, such as secondary glaucoma, injury of corneal endothelial cells and vitreoretinopathy when the dislocated IOL falls into the vitreous cavity. Whether IOL repositioning or IOL exchange is preferable for treating IOL dislocation remains controversial [14]. Here we meta-analyzed the available literature in English and Chinese on this question, and our results suggest that IOL exchange is superior to repositioning in terms of postoperative SE, while IOL repositioning is associated with lower incidence of anterior vitrectomy and a decreased trend of CME. Neither procedure significantly affects best corrected visual acuity and the incidence of IOL redislocation.

We found that pseudoexfoliation is a particularly strong risk factor for late in-the-bag IOL dislocation after cataract surgery; it was mentioned in 8 [9,11,14,15,19,20,22,24] of the 14 studies included in our review. Pseudoexfoliation can lead to zonular injury and progressive anterior capsulorhexis contraction. This alters the position of the IOL-bag complex, ultimately dislocating it [12,32,33]. Other risk factors for IOL dislocation included trauma [7,9,11,14,18,21], high myopia [11,14,24], previous intraocular surgery [14,18–20], capsulotomy or capsular rupture [7,11,18–20,23], uveitis [9,10,14], Marfan’s syndrome [24], retinitis pigmentosa [5].

Previous studies have reported that both IOL repositioning and exchange can improve BCVA significantly, and our meta-analysis suggested that the two procedures show similar efficacy for improving BCVA. Unfortunately, few studies reported data on uncorrected visual acuity, which is an important parameter for comparing the two treatments.

Pooled analysis of nine studies revealed no significant differences between IOL repositioning and exchange in incidence of IOL redislocation(P = 0.11).Although refixating the dislocated IOL is more difficult than implanting a new IOL, especially given the fact that the selection of suture position and operation technique are highly restricted, the two methods share the same stability if a IOL was sutured on sclera or iris successfully. Recently, a retrospective single-surgeon study of 118 eyes reported that scleral fixation sutures with 10–0 polypropylene provide excellent long term fixation of posterior chamber IOLs, resulting in suture breakage in fewer than 0.5% of cases for periods of 24 years and longer [34]. Of course, regardless of whether the surgeon is suturing a dislocated IOL or a newly implanted one, several factors influence the stability of the sutured IOL, including fixation technique, suture type, and knot stability. Experienced fixation technique and knot technique of operators may contribute to lower incidence of IOL redislocation, and 10–0 polypropylene suture and the knot technique requiring 2 separate sutures in one knot seems to be an ideal choice to keep knot stability [34,35,36].

Extracting a dislocated IOL or complex requires extensive incision, and the surgery can severely affect the vitreous and retina, leading to CME and anterior vitrectomy. Of the seven studies that reported CME incidence after surgery, one (Sarrafizadeh et al.2001) reported high incidence after each procedure. When we excluded patients who had undergone age related macular degeneration before surgery, we found that the incidence of CME tended to be lower among patients who underwent IOL repositioning than among those who underwent IOL exchange, although the difference was not significant(P = 0.06). Similarly, our meta-analysis revealed that repositioning was associated with significantly lower incidence of anterior vitrectomy. Therefore, IOL repositioning may be preferable for patients at high risk of CME, such as those with diabetes and high myopia.

Two studies [7][1] reported postoperative SE, and our pooled analysis revealed lower postoperative SE in the IOL exchange group, implying a higher rate of independence from corrective lenses in this group. This may reflect that, during IOL repositioning, the new position of the IOL nearly always differs from its initial position. In addition, the suture may loosen with time. A change of only 1 mm in anterior chamber depth corresponds to a 1.50D change in refraction [37]. In the IOL exchange procedure, accurate optical biometry can be performed before surgery, allowing the clinician to choose an appropriate refractive diopter according to the target position of the new IOL, allowing the clinician to precisely predict postoperative SE.

Pooled analysis of two studies [7][17] showed similar SIA incidence after the two procedures, with a non-significant tendency toward higher incidence after IOL exchange. A possible reason is that replacing a dislocated complex with a new IOL requires making a larger incision. This meta-analysis should be interpreted with caution, since one study [17] involved ab externo suture loop closed system fixation technique in the case of repositioning surgery and scleral pocket arcuate incision in the case of exchange surgery, whereas the other study [7] involved a limbal incision in the case of repositioning surgery, and a corneal incision with scleral incision in the case of exchange surgery. Comparison of these two studies suggests the possibility that scleral incision decreases risk of SIA, which should be examined in future work.

Overall, the results of our meta-analysis should be interpreted with caution in light of several limitations. One is that only 14 studies were involved, and they featured relatively small samples (1,082 eyes) with limited follow-up. Data from the included retrospective cases series were not adjusted through multivariate analysis. Nevertheless, most studies received high mean quality scores, even though only one was an RCT and the others were retrospective cases series. The fact that we included only full-length studies published in English or Chinese may increase risk of publication bias. In addition, the studies involved numerous surgeons who employed different operating techniques involving variations in fixation position, tips used, and incision sizes.

## Conclusion

When treating IOL dislocation, the two procedures of IOL repositioning and IOL exchange have similar efficacy in achieving postoperative BCVA and similar incidence of IOL redislocation, while IOL exchange may be associated with better uncorrected visual acuity. On the other hand, IOL repositioning is associated with lower incidence of anterior vitrectomy and possibly lower incidence of CME. In addition, IOL repositioning appears to be associated with lower of SIA, but this should be confirmed in future work. While the two procedures are associated with similar incidence of other complications, IOL repositioning is more cost-effective since there is no need for a new IOL implantation.

## Supporting information

S1 File Table PRISMA 2009 checklist. (DOC)

S2 File PRISMA 2009 flow diagram. (DOC)
