# Supplementary material for: Surgical management of intraocular lens dislocation: A meta-analysis
Source: PLoS One. 2019 Feb 20;14(2):e0211489. doi: 10.1371/journal.pone.0211489 (PMC6382138; doi:10.1371/journal.pone.0211489)
Supplement: S2 File — (DOC) [file pone.0211489.s002.doc]

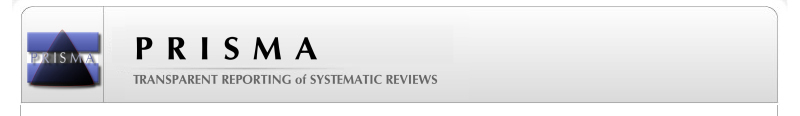
**PRISMA 2009 Flow Diagram**

**Screening**

**Included**

**Eligibility**

**Identification**

Records identified through whole database (n =4332)

Records screened(n=48)

Records excluded after duplication and reviewing title and abstract
(n = 4284)

Full-text articles assessed for eligibility (n = 15)

Full-text articles excluded
(n =33), for unqualified study setting(n=12), no available data (n=12), no appropriate intervention approaches we focus on(n=7) and other languages(n=2)

Studies included in qualitative synthesis（meta-analysis）
(n =14)

Studies included in quantitative synthesis (meta-analysis)
(n =14)
